# Adipose Gene Expression Prior to Weight Loss Can Differentiate and Weakly Predict Dietary Responders

**DOI:** 10.1371/journal.pone.0001344

**Published:** 2007-12-19

**Authors:** David M. Mutch, M. Ramzi Temanni, Corneliu Henegar, Florence Combes, Véronique Pelloux, Claus Holst, Thorkild I. A. Sørensen, Arne Astrup, J. Alfredo Martinez, Wim H. M. Saris, Nathalie Viguerie, Dominique Langin, Jean-Daniel Zucker, Karine Clément

**Affiliations:** 1 INSERM, Nutriomique U872, Paris, France; 2 Centre de Recherche des Cordeliers, Pierre and Marie Curie University, UMR S 872, Paris, France; 3 Université Paris Descartes, UMR S 872, Paris, France; 4 Laboratoire d'Informatique Medicale and Bio-Informatique (LIM&BIO) EA3969, Paris Nord University, Bobigny, France; 5 Centre for Health and Society, Institute of Preventive Medicine, Copenhagen University Hospital, Copenhagen, Denmark; 6 Department of Human Nutrition, Faculty of Life Sciences, University of Copenhagen, Copenhagen, Denmark; 7 Department of Physiology and Nutrition, University of Navarra, Pamplona, Spain; 8 Department of Human Biology, NUTRIM, Maastricht University, Maastricht, The Netherlands; 9 Inserm U858, Institut de Médecine Moléculaire de Rangueil, Laboratoire de recherches sur les obésités, Toulouse, France; 10 Institut Louis Bugnard, Université Paul Sabatier, IFR31, Toulouse, France; 11 Centre Hospitalier Universitaire (CHU) de Toulouse, Laboratoire de biochimie, Institut Fédératif de Biologie de Purpan, Toulouse, France; 12 Assistance Publique-Hôpitaux de Paris (AP-HP), Pitié Salpêtrière Hospital, Department of Nutrition and Endocrinology, Centre de Recherche en Nutrition Humaine Ile de France (CRNH, Idf), Paris, France; Minnesota State University Mankato, United States of America

## Abstract

**Background:**

The ability to identify obese individuals who will successfully lose weight in response to dietary intervention will revolutionize disease management. Therefore, we asked whether it is possible to identify subjects who will lose weight during dietary intervention using only a single gene expression snapshot.

**Methodology/Principal Findings:**

The present study involved 54 female subjects from the Nutrient-Gene Interactions in Human Obesity-Implications for Dietary Guidelines (NUGENOB) trial to determine whether subcutaneous adipose tissue gene expression could be used to predict weight loss prior to the 10-week consumption of a low-fat hypocaloric diet. Using several statistical tests revealed that the gene expression profiles of responders (8–12 kgs weight loss) could always be differentiated from non-responders (<4 kgs weight loss). We also assessed whether this differentiation was sufficient for prediction. Using a bottom-up (i.e. black-box) approach, standard class prediction algorithms were able to predict dietary responders with up to 61.1%±8.1% accuracy. Using a top-down approach (i.e. using differentially expressed genes to build a classifier) improved prediction accuracy to 80.9%±2.2%.

**Conclusion:**

Adipose gene expression profiling prior to the consumption of a low-fat diet is able to differentiate responders from non-responders as well as serve as a weak predictor of subjects destined to lose weight. While the degree of prediction accuracy currently achieved with a gene expression snapshot is perhaps insufficient for clinical use, this work reveals that the comprehensive molecular signature of adipose tissue paves the way for the future of personalized nutrition.

## Introduction

Personalized nutritional and medical intervention is rapidly gaining more interest as general population-based recommendations continue to produce unclear and contradictory results [1]–[4]. As such, the counsel and treatment of a complex disease like obesity requires a more fine-tuned and individual approach. Gene expression profiling has been positioned as a potential method for the identification of novel predictors of disease etiology, outcome, occurrence of co-morbidities and responsiveness to dietary intervention. While microarray analyses have been successfully used to classify and/or predict disease state or outcome in oncology [5]–[10], no examples currently exist in which gene expression has been used to predict changes in weight after nutritional or clinical intervention in obese individuals. Rather, the many microarray studies performed using obesity models have repeatedly demonstrated that microarray analysis can successfully differentiate between preadipocytes and adipocytes, the various body fat depots in humans, and the cellular response of adipocytes to exogenous compounds [11].

The complex biology of obesity suggests that using microarrays to identify genes capable of foreshadowing adipose tissue metabolism is an ambitious endeavor. This is primarily because of the large inter-individual differences in response to a given dietary intervention for which the origin of this variation remains unknown [12]. Indeed, common obesity does not occur because of a single dysfunctional gene, but rather through gene-gene and gene-environment interactions that will vary from one individual to another [13]–[15]. While most microarray work in obesity has focused on class comparison (i.e. comparing obese versus lean, adipocyte versus preadipocyte, etc.), the line between class comparison and class prediction is often blurred and results are frequently positioned to create interest in novel genetic markers for the purpose of disease prognosis and management [16], [17]. Yet there is an inherent limitation with such an approach as inferred models tends to ‘over-fit’ data [18]. Prior to claiming a gene or subset of genes as predictive, they must be confirmed in independent samples because often a predictor performs well only on the samples used to identify it. To circumvent this limitation, class prediction is best approached using supervised methods that extract information from a representative and randomly generated ‘training’ set under the guidance of a ‘teacher’, and then relate it to a ‘test’ set to confirm the validity of this extracted information. Only through such an approach can clinically relevant gene predictors be identified.

Selecting subjects from the Nutrient-Gene Interactions in Human Obesity-Implications for Dietary Guidelines (NUGENOB: http://www.nugenob.com) trial, we examined whether subcutaneous adipose tissue gene expression could be used to predict the amount of weight loss in an individual following the consumption of a low fat-hypocaloric diet. Subcutaneous tissue was used for prediction because of the relative ease and rapidity by which biopsies may be obtained. While little is known regarding the changes in adipose gene expression incurred following energy restriction with a low-fat, high-carbohydrate diet [19], [20], even less is known concerning the predictive capacity of human adipose gene expression profiles with regards to diet-induced weight loss. The aim of the present study was to assess whether a single gene expression snapshot prior to the 10 week consumption of a hypocaloric diet could be used to accurately predict whether an individual would lose weight or not. Through the use of a combination of statistical and supervised learning techniques, we demonstrate that the ability to distinguish between the expression profiles of dietary responders does not guarantee a high accuracy for class prediction. This is in contrast to the successful class comparison and prediction achieved with a publicly available cancer dataset [5]. These results are particularly relevant to the clinical field and applicable to those studying drug and nutritional responses in humans with nutritionally related diseases, such as obesity and its associated metabolic pathologies.

## Materials and Methods

### Subjects and study design

Subjects were participants in the European multi-centre NUGENOB study (www.nugenob.org), which was supported by the European Community. Informed consent was obtained from all subjects. Clinical investigations were approved by the ethical committees of each participating centres and were performed according to the Declaration of Helsinki. Subjects (n = 771) were randomly assigned to one of two similarly energy-restricted diets: a low-fat, high-carbohydrate diet (LF) or a moderate-fat, low-carbohydrate diet (MF); however, the present study involved only females from the LF group. The LF diet was designed to provide 600 kcal/day less than the individually estimated energy requirement based on an initial resting metabolic rate measurement and multiplied by 1.3. The macronutrient composition of the LF diet was 20–25%∶15%∶60–65% for fat:protein:carbohydrate, respectively. Subjects completed a 3-day weighed food record for two weekdays and one weekend day before the start of the dietary intervention and at the end of the 10-week diet. This was done to assess the habitual diets of the subjects and to estimate their compliance, respectively. Subjects also completed 1-day weighed food records during the second, fifth and seventh weeks of the intervention. During the dietary intervention the subjects either visited or had telephone contact with the dietician every week.

### Subject Selection for Prediction Analysis

**Figure 1:**
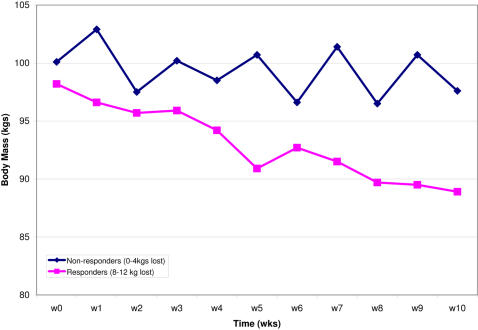
Weight loss curves during the 10 week hypocaloric diet. The two groups were defined as responders (i.e. subjects losing between 8–12 kgs) and non-responders (i.e. subjects losing less than 4 kgs). Weight was measured in at least 43 subjects at each weekly time point. Error bars represent the 95% confidence intervals (equal to 1.96 * standard deviation).

Subcutaneous adipose tissue biopsies were obtained for the majority of the 771 subjects participating in this dietary intervention study (both before and after the dietary intervention). An abdominal subcutaneous fat specimen (∼1 g) was obtained by needle aspiration under local anaesthesia after an overnight fast. Biopsies were washed and stored in RNA later preservative solution (Qiagen, Courtaboeuf, France) at −80°C until analysis. Total RNA was extracted using the RNeasy total RNA Mini kit (Qiagen). When accounting for both drop outs during the intervention study and those biopsies that produced total RNA that did not meet quality and quantity controls, 319 subjects were assessed for weight loss after 10 weeks and subsequently divided into two groups: ‘responders’ (i.e. subjects losing between 8–12 kg) and ‘non-responders’ (subjects losing <4 kg) (**Figure 1**). Twenty-seven female subjects were randomly selected from each group after careful matching based on weight, height, body mass index (BMI), waist/hip ratio, energy intake, fat, carbohydrate, protein and alcohol energy intakes at baseline (**Table 1**). Only total RNA from biopsies taken prior to the 10 week dietary intervention was used in the present study.

**Table 1:** Baseline characteristics of responders (8–12 kgs weight loss) and non-responders (<4 kgs weight loss) at T0.

Group	Non-responders	Responders
Number of Subjects	26	27
Age	34.0±10.0	37.7±8.5
Weight (kg)	100.9±15.6	96.4±14.3
BMI (kg.m^−2^)	37.8±5.9	35.6±5.1
FFM (kg)	54.5±5.9	54.4±4.4
FM (kg)	46.4±12.0	42.0±11.3
WHR	0.85±0.07	0.85±0.06
Triglycerides (µmol/L)	980±370	958±328
Free fatty acids (µmol/L)	552±161	509±111
Free glycerol (µmol/L)	130±108	108±65
Total cholesterol (mmol/L)	5.07±0.86	5.05±0.97
HDL-C (mmol/L)	1.24±0.37	1.27±0.28
LDL-C (mmol/L)	3.39±0.87	3.35±0.93
VLDL-C (mmol/L)	0.27±0.07	0.29±0.09
Insulin (µU/ml)	11.1±5.8	9.0±6.0
Leptin (ng/ml)	37.4±12.7	32.4±9.7
Cortisol (nmol/L)	221±119	227±117
Glucose (mmol/L)	5.11±0.39	5.23±0.33

Values are means±standard deviation. Values indicated are from the fasted state. FFM, fat free mass; FM, fat mass.

### Sample preparation and microarray analysis

Total RNA concentration and quality was confirmed using the Agilent 2100 Bioanalyzer (Agilent Technologies, Massy, France). 200 ng of total RNA from each sample was amplified and transcribed into fluorescent cRNA using Agilent's Low RNA Input Linear Amplification kit (Agilent Technologies, Massy, France). Cyanine-5 dye was incorporated into all samples, while an in-house obese reference pool was labeled with cyanine-3 dye. In brief, the in-house reference pool was created by mixing equal amounts of total RNA extracted from adipose tissue samples of subjects undergoing plastic surgery, as previously described [21]. Samples were hybridized to Agilent 44K whole human genome microarrays, which are comprised of over 41,000 unique 60-mer oligonucleotide human sequences and transcripts. Sample preparation, hybridization, and microarray washing were performed according to manufacturer's recommendations (Agilent Technologies, Massy, France). Arrays were scanned using a GenePix 4000A Scanner (Axon Instruments-Molecular Devices, Sunnyvale, CA).

### Differential Gene Expression Analysis

Fifty-four microarrays, corresponding to 27 responders and 27 non-responders, were performed; however, one of the microarrays in the non-responders group was of poor quality and therefore not considered further. For the remaining 53 arrays, background signal was not subtracted prior to the Loess normalization of log-transformed microarray data [22]. 14135 unique GeneIDs were present across 80% of the microarrays, representing 76.4% of the total genes on the 44K microarray platform. Differential gene expression, using a 5% false discovery rate (FDR), was assessed using the Significance Analysis of Microarrays (SAM) procedure (available at http://www-stat.stanford.edu/tibs/SAM/), a Fisher test and a paired Student's T-test. The three statistical approaches were used because of recent reports demonstrating that different algorithms can generate gene lists with differing degrees of predictive power [16], [17]. The Student's T-test score was generated using the mt.teststat function in the multtest package in ‘R’ (http://cran.r-project.org/src/contrib/Descriptions/multtest.html). In all analyses the threshold for statistical significance was p<0.05. Univariate analyses were performed using R software (available at http://www.r-project.org/). Multivariate analysis was performed using Umetrics SIMCA-P software (Umetrics AB, Umea, Sweden).

### Semi-quantitative real-time RT-PCR

Reverse transcription was performed with 0.5 µg of total RNA and random hexamer primers, according to manufacturer's instructions (Promega, Charbonnieres-les-Bains, France). Sybr® green primers were designed and validated for target specificity and amplification efficiency. Primer sequences for *Ptgds, Fmod, Ifi27, Qprt, Fam69B, Tmem132A, Esam, Cldn5,* and *Loc374491* are listed in supplementary materials (**Table S1**). RT-PCR amplification was performed using an ABI 7300 (Applied Biosystems, Foster City, CA, USA) with the following thermal cycling conditions: 2 min at 50°C, 10 min at 95°C, followed by 40 cycles of 95°C for 15 s and 60°C for 1 min for denaturation, annealing, and elongation. All samples were normalized to *18S* gene expression (18S rRNA Control kit, Eurogentec, Seraing, Belgium). Differences in gene expression were assesses using a two-tailed, homoscedastic Student's *t*-test.

### Prediction Analysis

Prediction analyses were performed using the following commonly used methods: Diagonal Linear Discriminant Analysis (DLDA) [23], K-Nearest Neighbour (KNN), Random Forest (RF) [24]–[27], and Support Vector Machines (SVM) [23], [24], [28], [29]. All algorithms were implemented within the R environment: the DLDA model is available in the *sma* package, KNN is in *class* package, RF is in the *randomForest* package and SVM in *Kernlab.* Standard parameters were used for all methods except SVM, where the optimal slack parameter was tuned using the *svmpath* package [30]. Only the 10592 GeneIDs for which no missing values were observed across all microarrays were used.

### Golub Leukemia Data

To assess the performance of our statistical tests, a human Affymetrix microarray dataset corresponding to 47 subjects with acute lymphoblastic leukaemia (ALL) and 25 subjects with acute myeloid leukaemia (AML), as previously described by Golub and colleagues [5], was downloaded from: http://wwwmaths.anu.edu.au/johnm/r/hddplot/. The ‘Golub’ cancer dataset profiled the expression of 7129 genes in 47 subjects with acute lymphoblastic leukemia (ALL) and 25 subjects with acute myeloid leukemia (AML) to develop a class predictor [5]. GeneChips were normalized using the median signal across all 72 microarrays. While the original dataset was comprised of three groups (AML, ALL T-type and ALL B-type), the present analysis created only two classes: AML and ALL. This was done in order to perform a comparable analysis to the present two-class dietary response study and to ensure that all methodologies used were performing as expected.

## Results

Gene expression profiling immediately before the 10-week consumption of a low-fat, high-carbohydrate diet (LF-diet) was performed to assess whether subjects could, *a priori*, be classified as hypo-caloric diet responders and non-responders. To test this hypothesis, we determined whether A) responders could be differentiated from non-responders based on microarray data, and B) whether these gene expression profiles could be used for class prediction (i.e. can one predict whether an individual will lose weight in response to dietary intervention or not).

### Differentiating Responders from Non-Responders

**Figure 2:**
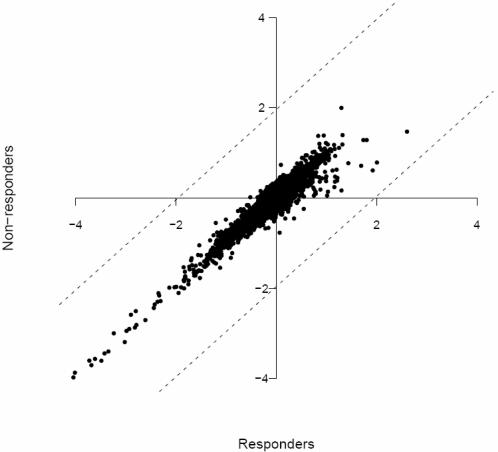
Distribution of the mean gene expression levels in responders and non-responders, computed from microarray measurements normalized with respect to the standard Gaussian distribution. Each spot represents the mean expression for a single gene. Dotted lines indicate the 95% confidence interval of the means (equal to 1.96 * standard deviation).

As illustrated in **Figure 2**, global gene expression was similar between dietary responders and non-responders. Nevertheless, using any of 3 different statistical approaches enabled us to identify gene sets differentiating the two populations. While a SAM analysis using a false discovery rate (FDR) of 5% did not identify any differences between the two populations, relaxing our selection criteria to a FDR of 8% identified 34 differentially expressed genes. The selection criteria for the two other analytical tests (Fisher test and Student's T-test) were arbitrarily set in such a manner as to select the top 100 genes that differentiated the two populations. In order to minimize spurious results that could be attributed to the varying sensitivity of the statistical tests and their ability to identify outliers (i.e. differentially expressed genes), we considered only those genes that were identified by all 3 statistical tests as candidate predictive genes (herein referred to as ‘predictors’). The following nine genes were identified as significantly increased in non-responders versus responders: prostaglandin D2 synthase (*Ptgds* : 1.6-fold), claudin 5 (*Cldn5* : 1.4-fold), fibromodulin (*Fmod* : 1.4-fold), interferon alpha-inducible protein 27 (*Ifi27* : 1.4-fold), quinolinate phosphoribosyltransferase (*Qprt* : 1.3-fold), family with sequence similarity 69 B (*Fam69b* : 1.3-fold), transmembrane protein 132A (*Tmem132A* : 1.2-fold), endothelial cell adhesion molecule (*Esam* : 1.2-fold), and TPTE/PTEN homologous inositol lipid phosphatase pseudogene (*LOC374491* : 1.1-fold). Statistical significance for *Ptgds*, *Cldn5*, *Qprt*, and *Tmem132A* was confirmed by real-time RT-PCR (p<0.05; **Table 2**); and while directional concordance was achieved for *Fmod*, *Ifi27*, *Fam69b* and *Esam*, changes in expression were not statistically significant. Suitable primers for *LOC374491* could not be designed; therefore this gene was not confirmed by real-time RT-PCR. Nevertheless, these 9 genes were deemed suitable candidates to assess their capacity to predict weight loss.

**Table 2:** Validation of 8 predictors by real-time RT- PCR in comparison to microarray results.

Transcript Name	FC by Microarray (FDR = 8%)	FC by real-time RT-PCR	*P*-value for real-time RT-PCR
TMEM132A	1.2	2.6	0.008
QPRT	1.3	2.1	0.015
CLDN5	1.4	2.3	0.015
PTGDS	1.6	1.9	0.035
ESAM	1.2	2.2	0.126
FMOD	1.4	2.3	0.159
FAM69B	1.3	2.1	0.176
IFI27	1.4	1.4	0.182

Of the 8 genes examined by real-time RT-PCR (normalized to 18S rRNA), all of them were in directional concordance with microarray results; however, only 4 of them were statistically significant (*p*<0.05). FC (fold change) measurements represent non-responder vs. responder, where a positive FC indicates the transcript is more highly expressed in non-responders. FDR, false discovery rate.

**Figure 3:**
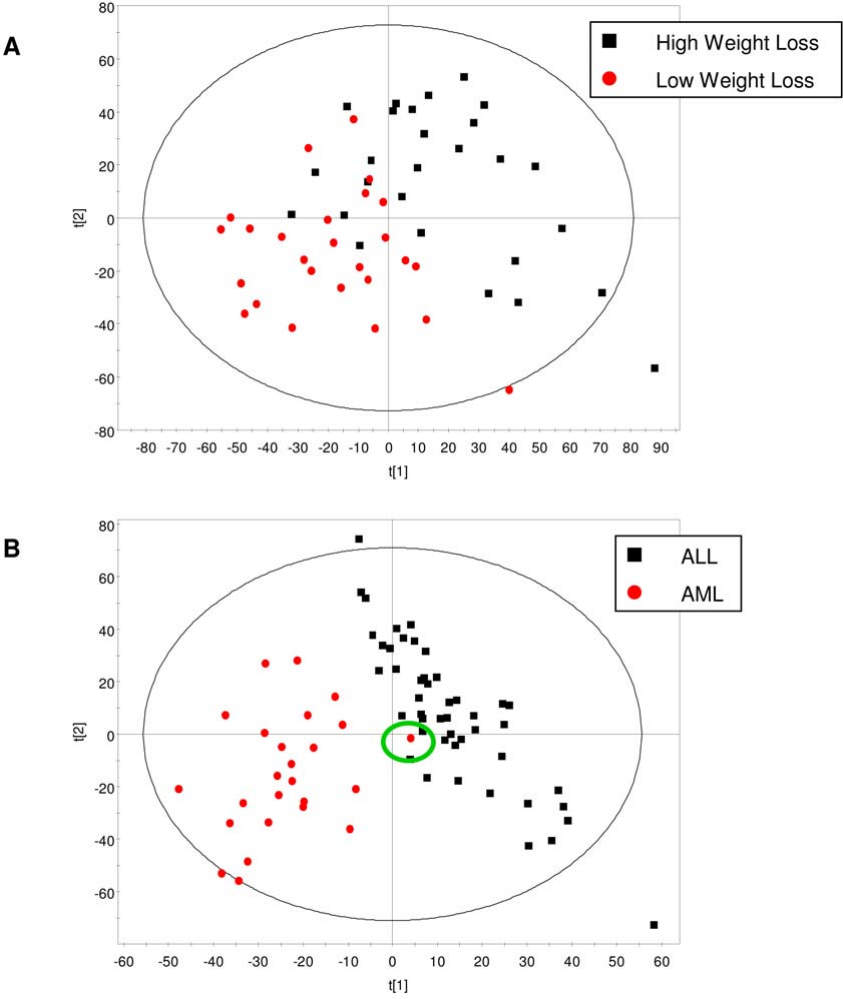
A. Differentiating populations by PLS-DA. Global gene expression analysis in sub-cutaneous tissue reveals a separation trend between dietary responders (black squares) and non-responders (red circles); however, there is a significant overlap between the two populations (R^2^ = 0.547 and Q^2^ = −0.096). B. ALL patients (black squares) can be clearly separated from AML patients (red circles), apart from a single patient (identified by the green circle) (R^2^ = 0.795 and Q^2^ = 0.622). R^2^ explains the cumulative variation of the first two components and Q^2^ indicates the variation explained by the model according to cross validation. Only a Q^2^>0.5 indicates a good model.

In order to determine whether global gene expression profiles could permit responders and non-responders to be distinguished, partial least squares discriminant analysis (PLS-DA) was performed. **Figure 3A** demonstrates that while a trend regarding the separation of the two groups using subcutaneous adipose tissue gene expression profiles exists, there is some overlap between the two populations (R^2^ = 0.547 and Q^2^ = −0.096, where R^2^ explains the cumulative variation of the first two components and Q^2^ indicates the variation explained by the model according to cross validation). In contrast to our adipose gene expression data, **Figure 3B** clearly illustrates that the Golub cancer dataset can be differentiated by PLS-DA, apart from a single outlier, explained below (R^2^ = 0.795 and Q^2^ = 0.622). Although the first two principal components explain a significant amount of the variation in both datasets, only the Golub model performs well when cross-validated.

### Predicting Clinical Responders from Non-Responders

Because the two obese groups could be differentiated as previously described, we employed several supervised machine learning algorithms to address whether this differentiation was sufficient to establish a robust classifier to identify responders and non-responders. Classification was assessed using support vector machine (SVM), random forest (RF), K-nearest neighbour (KNN), and diagonal linear discriminant analysis (DLDA) using a ten times 10-fold cross validation approach. Simultaneously, we examined the prediction accuracy of robust classifiers using a bottom-up analysis and a top-down analysis [16]. Whereas a bottom-up analysis uses all gene expression data in a ‘black-box’ approach, a top-down analysis uses existing biological knowledge to build a classifier (i.e. differentially expressed genes and/or molecular pathways).

A training model for each classification algorithm was established using a cross-validation method, i.e. 9/10^th^ of the cohort was used to establish a prediction model (training set) and the remaining 1/10^th^ was used as a test set [31]. This process was repeated 10 times for each algorithm to assess variability in prediction accuracy. In all cases, prediction accuracy was compared to the ‘no information’ algorithm result that places all subjects in the majority class (i.e. for adipose tissue gene expression data = 51%; for Golub cancer data = 65%), where a value above the ‘no information’ result was considered an improvement in prediction accuracy. Using the entire microarray dataset (i.e. 10592 genes), a bottom-up analysis led to insignificant improvements in prediction accuracies versus the ‘no information’ algorithm (**Table 3**). KNN performed best, giving a prediction accuracy of 61.1%±8.1%. SVM, RF, and DLDA were able to predict 45.9%±5.7%, 52.9%±11.3%, and 52.6%±8.2%, respectively. In contrast, using the entire Golub cancer dataset (processed identically to our complete adipose gene expression dataset), tumor prediction accuracy soared to 98.6%±0.0%, 96.9%±2.2%, 92.0%±2.9%, and 89.0%±4.1% using SVM, RF, KNN, and DLDA, respectively. The single outlier identified by PLS-DA (**Figure 3B** **–green circle)** was the only subject in the Golub dataset to be consistently misclassified by all supervised learning methods.

**Table 3:** Prediction accuracies using ten times 10-fold cross validation with different gene subsets.

*Data Used*	*Learning Models*			
	*SVM*	*RF*	*KNN*	*DLDA*
All Genes *(10592 genes)*	45.9%±5.7	52.9%±11.3	61.1%±8.1	52.6%±8.2
Fisher Analysis using a Training Set *(Top 100 genes)*	49.3%±11.3	53.4%±7.6	53.0%±6.6	54.2%±5.4
Student's T-Test Analysis using a Training Set *(Top 100 genes)*	45.7%±10.5	51.6%±5.7	47.9%±13.9	56.6%±6.1
SAM Analysis with an FDR = 8% *(34 genes)*	70.2%±5.7	74.9%±8.1	73.7%±4.5	80.9%±2.2
Fisher Analysis *(Top 100 genes)*	67.8%±11.2	69.8%±4.6	54.9%±6.3	70.6%±4.0
Student's T-Test Analysis *(Top 100 genes)*	77.8%±7.9	71.0%±4.9	74.0%±4.9	78.8%±2.8
Combined SAM, Fisher, & Student's T-Test *(9 genes)*	73.0%±7.1	68.5%±5.3	66.5%±4.0	69.2%±3.2
Golub Cancer Data *(7129 genes)*	98.6%±0.0	96.9%±2.2	92.0%±2.9	89.0%±4.1

SVM, support vector machine; RF, random forest; KNN, K-nearest neighbour, DLDA, diagonal linear discriminant analysis; SAM, significance analysis of microarrays; FDR, false discovery rate. Values represent mean±standard deviation corresponding to the 95% confidence interval.

In an attempt to improve our prediction accuracy, we examined gene sets obtained by Fisher and Student's T-tests. As the goal is to identify reliable predictors that can be used to screen whether new patients will lose weight with a hypocaloric diet or not, predictors need to be identified in a training set and not the entire dataset, i.e. valid predictors should consistently differentiate responders from non-responders in all possible comparisons, and not be dependent on a single comparative analysis. Therefore, the Fisher and Student's T-tests were performed using only gene expression data in the training set (corresponding to 9/10^ths^ of dataset) to identify the top 100 differentially expressed genes. These 100 differentially expressed genes were then subsequently tested in the remaining 1/10^th^ of the dataset. Neither of these tests led to improvements in prediction accuracies (**Table 3**).

Finally, using a purely top-down approach, the results obtained from the SAM, Fisher, and Student's T-test analyses were examined to determine whether these lists of differentially expressed genes could serve as reliable ‘predictors’. These gene lists can be found in the supplementary materials (**Table S2**). In contrast to the previous Fisher and Student's T-test analyses, these different gene sets were obtained using all 53 microarrays and not a cross-validation method. The 34 differentially expressed genes identified by SAM led to prediction accuracies of 70.2%±5.7%, 75.0%±8.1%, 73.7%±4.5%, and 80.9%±2.2% for SVM, RF, KNN, and DLDA, respectively. While prediction accuracies improved overall with a top-down approach, neither the top 100 genes identified with a Fisher test, the top 100 genes identified with a Student's T-test, nor the 9 common genes performed better than the 34 genes identified by SAM (**Table 3**).

## Discussion

The ability to predict whether an individual will respond successfully to dietary intervention with a significant weight loss clearly has important clinical ramifications. Indeed if a clinician could, *a priori*, know whether a patient's health status will improve in response to the consumption of a given diet then disease management will be profoundly modified in many aspects. The analysis of gene expression has been positioned, in large part because of its success in the field of oncology, as one means by which an individual's response to an intervention could be predicted. The present study revealed that while a single comprehensive snapshot of gene expression immediately prior to the 10-week consumption of a hypocaloric diet can differentiate responders from non-responders, at this stage it is insufficient for the accurate class prediction required for clinical use.

Studies in the oncology field suggest that classifying tumors by microarray analysis can be achieved, but despite these encouraging findings there is, up to now, no evidence that this approach can be useful in the study of nutrition related diseases such as obesity. When one considers that adipose tissue metabolism is regulated by not only an individual's genetic make-up, but also the obesogenic environmental factors an individual is exposed to (e.g. diet, physical activity, gut microbiota, viruses, etc.), then one must clearly consider the individual (or subset of individuals) to identify eventual responders from non-responders [13], [32]. As previously reported, significant inter-individual differences in response to dietary interventions demonstrate that weight loss is governed by both genetic and lifestyle components [19], [33], [34]. Indeed, class prediction would be simplified considerably if the aforementioned diseases arose purely because of gene dysfunction, rather than having both a genetic and environmental component.

Profiling subcutaneous adipose gene expression in 53 human subjects participating in the NUGENOB trial prior to the start of a 10-week hypo-caloric diet has revealed that responders (subjects losing between 8–12 kg) can be distinguished from non-responders (subjects losing less than 4 kg); however, the differences are minimal and do not cleanly separate, as illustrated by multivariate analysis. This was in contrast to the distinct separation of AML versus ALL subjects seen in the Golub cancer dataset. Thus, while differences in adipose gene expression profiles were identified (using SAM, Fisher and Student's T-test analyses), we hypothesize that the considerable overlap seen with PLS-DA confounds our ability to predict class (61.1% accurate at best using a bottom-up analysis). Furthermore, the low accuracies suggest that there are no dominant predictors within the gene expression dataset analyzed in this study. This is dramatically different from the Golub cancer dataset were we observed a nearly perfect class prediction (98.6% accuracy). Even though prediction accuracies were improved when we used a top-down analysis using differentially expressed gene sets, we still could not identify a classifier that performed as well as the classifier inferred from the Golub dataset. It is important to note that the differentially expressed gene sets used for the top-down analysis were generated using all 53 microarrays. Thus, even though we could achieve upwards to 80% prediction accuracy, the predictive power of these gene subsets requires validation in new subjects as they risk being specific to only the subjects used in the present study and therefore of little clinical relevance [10], [17]. This finding also highlights an important difference between our data and the Golub dataset–namely that we have attempted to predict an individual's response rather than classify an individual's disease state. As such, the present study implies that classifying tumor type might be less complex than predicting an individual's response to an intervention. Nevertheless the Golub dataset was perfectly suited to confirm that our prediction methodologies were functioning correctly. Therefore we believe that predicting a response to diet using a single snapshot of global gene expression prior to an intervention is not sufficient to identify responders from non-responders.

Examples exist in which an individual or subset of predictive genes have been proposed; however, these studies have performed class comparison rather than class prediction. For example, Tseng and colleagues performed microarray analysis of brown preadipocytes and postulated insulin receptor substrates and necdin as predictive genes for differentiation [35]. And more recently, Koza *et al*. profiled adipose tissue gene expression and revealed that mice with low or high body fat gain could be distinguished prior to the consumption of a high fat diet [36]. While these studies may suggest that identifying predictive genes using a gene expression snapshot is possible, there are several critical differences between our work and these two aforementioned studies. Firstly, neither of these studies used humans. As such, using genetically identical mice eliminates a major confounder present in human studies. Secondly, neither study used supervised methods to identify predictors; rather, they both used statistical approaches to identify differentially regulated genes. As such, the actual predictive value of their candidates was not assessed. Thus, while these previous studies provide molecular insight into fat mass expansion, they have yet to definitely demonstrate that their molecular targets are clinically-reliable predictors.

To the best of our knowledge, the use of gene expression to predict a response to dietary intervention in humans has not been previously performed; however, examples do exist in which the efficacy of anti-obesity drugs (sibutramine and orlistat) have been explored. Drug efficacy for an individual subject was determined by assessing weight loss in the first 3 months of treatment [37], [38]. Subjects reaching a given threshold for weight loss in the first months of treatment were predicted to benefit from the long term administration of these anti-obesity drugs. Relating these findings to our microarray results suggests that predicting classes in obesity may be improved by studying changes in gene expression once dietary intervention has begun (rather than single time points). Such an approach would allow one to assess how the biological system responds once it has been challenged with an exogenous factor, and thereby possibly improve class prediction.

In conclusion, microarrays can provide sound molecular insight into the biological mechanisms underlying adipose tissue metabolism; however, their potential in a clinical context to assist in the optimized nutritional counseling of obese individuals remains in its infancy. Nevertheless, alternate strategies to maximize the wealth of information on microarrays are being explored and suggest that predicting diet response for an individual will become more increasingly more accurate in the near future.

## Supporting Information

Table S1 Primer sequences for transcripts validated by real-time reverse-transcriptase PCR.
(0.02 MB XLS)

Table S2 Gene lists used for the top-down analyses. The gene lists indicate the 34 genes identified with SAM and the top 100 genes identified by Fisher or Student's t-test analyses. SAM, significance analysis of microarrays; FDR, false discovery rate.
(0.04 MB XLS)
